# Regional Inequalities and Influencing Factors of Residents’ Health in China: Analysis from the Perspective of Opening-Up

**DOI:** 10.3390/ijerph191912069

**Published:** 2022-09-23

**Authors:** Guozhen Gao, Jinmiao Hu, Yuanyuan Wang, Guofeng Wang

**Affiliations:** 1 Faculty of International Trade, Shanxi University of Finance and Economics, Taiyuan 030006, China; 2 School of Public Administration, Shanxi University of Finance and Economics, Taiyuan 030006, China

**Keywords:** opening-up, residents’ health, regional inequalities, Difference-in-Difference model

## Abstract

While opening-up promotes regional economic development, its impact on the residents’ health level cannot be ignored. Based on provincial data of China from 2009 to 2020, the Gini Coefficient and Theil Index are used to analyze the regional inequalities in residents’ health in China. The Difference-in-Difference model is constructed to study the impact of China’s opening-up policies and other factors on residents’ health. The results show that, firstly, the health levels of Chinese residents have steadily improved and regional inequalities have been gradually narrowing. Secondly, the Belt and Road Initiative has significantly improved the residents’ health along the route, while the Pilot Free Trade Zone, which is another important opening-up policy in China, has had an inhibitory effect on the health of residents. Thirdly, it is proven that the Belt and Road Initiative improves the health of residents in provinces along the route by increasing the degree of opening-up and improving the regional environmental quality. This study will support and advance the UN’s Sustainable Development Goals (SDGs), especially SDG3 (Good Health and Well-being) and SDG10 (Reduced Inequalities).

## 1. Introduction

While opening-up promotes regional economic development, its impact on the soft environment of regional development, such as the health level of residents, cannot be ignored. The extensive opening-up model may cause irreversible damage to regional climate conditions and the health of residents. Opening-up should not be done at the cost of damage to climate conditions or the health of residents. The health of residents should be prioritized in strategic development, since promoting the coordinated development of regional health, climate and economy is an inevitable requirement for the sustainable development of human society. Health is the foundation of human survival and development. The increase in quality of life and the sustainable development of society depends on the improvement of residents’ health levels. Since the 18th National Congress of the Communist Party of China, China has always practiced the important decision of “promoting the construction of a healthy China in an all-round way” and implemented the Healthy China 2030 strategy and Healthy Cities Pilot Project. It is hoped that by integrating health into various policies, the health level of Chinese residents will be improved and the regional gap of it will be narrowed.

The influencing factors of residents’ health is a hot topic both in China and abroad. From a global perspective, based on the data of 185 countries, it has been proven that the level of corruption would significantly influence the physical health (like life expectancy and Mortality rate) and mental health (like happiness) of residents [1]. It has been found that the heat waves in France have caused more excess mortality and years-of-life loss, and the population has experienced restricted activity [2]. In addition, the relationship between economic development and residents’ health has been detected [3,4,5,6]. Considering the influence of opening-up on residents’ health, it has been confirmed that the impact of foreign direct investment could have a negative impact on residents’ health, especially in the most developed countries [7].

In terms of research focused on China, from the perspective of environmental or climate-influencing factors, it has been proven that there is a significant negative relationship between the use of clean energy and maternal mortality in China [8]. The synergy effect of climate policies in China on public health and climate conditions has concluded that climate policies implemented in China have not only materially improved air quality but benefited health as well [9]. In view of the choice of cooking fuel in rural China, there is a significant and positive relation between utilizing non-solid cooking fuels and residents’ ability to deal with daily activities [10]. From the perspective of social influencing factors, in view of retirement, it has been confirmed that retirement could increase weight and Body Mass Index (BMI) among Chinese men and this effect is more obvious for men with low education levels [11]. Considering the policy of health insurance, the integration of rural and urban social health insurance systems could increase residents’ inpatient care utilization by middle-aged and older residents in rural areas, while it has not played a role in the health of residences [12]. As for transportation accessibility, it has been found that access to a high-speed train in China has not only improved the health of residents but reduced health inequality between urban and rural areas [13]. Focused on income, it has been proven that the increase in minimum wage can improve the health level of rural workers and workers over 35 years old [14].

The Belt and Road Initiative and the Pilot Free Trade Zone are important policies in China to promote opening-up in recent years. The existing literature has fully confirmed their roles in promoting the growth of regional economic scale and improving the regional environment [15,16,17,18]. However, there are scarce studies conducted on the possible impact of opening-up policies on residents’ health. There is also a lack of research that comprehensively considers the regional inequalities and influencing factors of the health level of residents. Against the backdrop of the frequent occurrence of global non-traditional security issues such as climate change, major public health emergencies and resident living conditions deterioration, analyzing the regional inequalities and influencing factors of the health level of Chinese residents and effectively identifying the impact and mechanism of opening-up on the health level of residents in China will help human beings globally to find appropriate solutions in the face of an unprecedented health crisis. In addition, this study will support and advance the UN’s Sustainable Development Goals (SDGs), especially SDG3 (Good Health and Well-being) and SDG10 (Reduced Inequalities).

## 2. Materials and Methods

### 2.1. Calculation of Residents’ Health Level

The residents’ health levels are mainly measured by indicators such as mortality, morbidity and life expectancy. Limited by data availability, there is a lack of annual provincial data on average life expectancy. Therefore, referring to Zhao et al. [19], based on the entropy method, the child (in perinatal) mortality rate, maternal mortality rate, infectious diseases morbidity rate and gynecological diseases detection rate are selected to calculate the residents’ health level. The mortality rate, morbidity rate and disease detection rate are all negative indicators, that is, the higher the value, the lower the health level of residents. In order to comprehensively measure the regional health level of residents, the data is first processed by reverse standardization, then the entropy method is used to determine the weight of each indicator and finally, the comprehensive score is obtained by linear weighting. Therefore, the Health Index of residents in each province is calculated to represent the regional health level of residents (Table 1).

(1) $${Health}_{i}=\sum_{t=1}^{4}h_{it}\times \omega_{it}$$

where ${Health}_{i}$ represents the residents’ health level of province i, $h_{it}$ represents the reverse normalized value of the tth health indicators of province i, $\omega_{it}$ represents the weight of the tth health indicators of province i.

### 2.2. Measurement of Regional Inequalities in Residents’ Health Level

#### 2.2.1. Gini Coefficient

The regional inequalities and changing trends of the health level of Chinese residents can be measured by Gini Coefficient [20]. It is assumed that there are n provinces included in the sample, $w_{i}$, ${Health}_{i}$ and $p_{i}$ represent the share of the Health Index of residents, the Health Index of residents and the province frequency of province i, respectively. After sorting the Health Index of all provinces in ascending order, the Gini Coefficient of residents’ health level in each year can be calculated as follows.

(2) $$Gini=1-\sum_{i=1}^{n}p_{i}(2\sum_{k=1}^{i}w_{k}-w_{i})$$

#### 2.2.2. Theil Index

The overall inequalities of the health level of Chinese residents can be further decomposed into the inequalities within the three major areas and between the three major areas according to the East, Central and West areas by Theil Index [19,21].

(3) $$Theil=\sum_{i=1}^{n}H_{i}\ln(nH_{i})={Theil}_{WA}+{Theil}_{BA}$$

(4) $${Theil}_{WA}=\sum_{i=1}^{n_{e}}H_{i}\ln(n_{e}\frac{H_{i}}{H_{e}})+\sum_{i=1}^{n_{c}}H_{i}\ln(n_{c}\frac{H_{i}}{H_{c}})+\sum_{i=1}^{n_{w}}H_{i}\ln(n_{w}\frac{H_{i}}{H_{w}})$$

(5) $${Theil}_{BA}=H_{e}\ln(H_{e}\frac{n}{n_{e}})+H_{c}\ln(H_{c}\frac{n}{n_{c}})+H_{w}\ln(H_{w}\frac{n}{n_{w}})$$

where: Theil is Theil Index, which represents the overall inequalities of residents’ health level in China; ${Theil}_{WA}$ is the Theil Index within three major areas, which represents the inequalities within three major areas; ${Theil}_{BA}$ is the Theil Index between three major areas, which represents the inequalities between three major areas; n is the number of provinces; $n_{e}$, $n_{c}$ and $n_{w}$ represents the number of provinces in East, Central and West areas, respectively; $H_{i}$ is the ratio of Health Index of province i to the national average level; $H_{e}$, $H_{c}$ and $H_{w}$ represent the ratio of Health Index of the East, Central and West areas to the national average level, respectively.

### 2.3. Difference-in-Difference Analysis of Factors Influencing Residents’ Health Level

The Belt and Road Initiative and the Pilot Free Trade Zone are important measures taken by China to deepen its opening-up in recent years. While promoting regional economic development, opening-up may have an impact on the soft environment (such as health) for regional development. Therefore, based on the analysis of regional inequalities in residents’ health levels in China, the Difference-in-Difference model is constructed to identify the impact of the opening-up policies and other factors on the health level of Chinese residents.

(6) $${Health}_{ij}=\beta_{0}+\beta_{1}{Treat}_{i}\times post_{j}+\gamma X_{ij}+\alpha_{i}+\delta_{j}+\varepsilon_{ij}$$

where $Health_{ij}$ represents the residents’ health level of province i in year j; treatment group dummy variable $Treat_{i}=1$ represents the province i is the province along the route of the Belt and Road Initiative, which includes 17 provinces specifically (Determined by the key provinces planned by the *Vision and Actions on Jointly Building Silk Road Economic Belt and 21st-Century Maritime Silk Road*. Except for the missing data of Tibet, including Xinjiang, Shaanxi, Gansu, Ningxia, Qinghai, Inner Mongolia, Heilongjiang, Jilin, Liaoning, Chongqing, Guangxi, Yunnan, Shanghai, Fujian, Guangdong, Zhejiang and Hainan); treatment time dummy variable $post_{j}=1$ represents the year j has already belonged to the year when the Belt and Road Initiative was proposed or the later (that is, from 2013 to 2020, in September and October 2013, Chinese President Xi Jinping proposed the cooperation initiatives to build the “New Silk Road Economic Belt” and the “21st Century Maritime Silk Road”, respectively); $X_{ij}$ is the control variable groups; $\alpha_{i}$ is the provincial fixed effect; $\delta_{j}$ is the year fixed effect; $\varepsilon_{ij}$ is the perturbation term; $\beta_{1}$ represents the net impact of the Belt and Road Initiative on the residents’ health level of provinces along the route.

In terms of control variable groups, referring to the existing literature about the analysis of the influencing factors of residents’ health level [19,22], a total of 9 control variables in the following 8 categories are added to further analyze the factors influencing the health level of residents in China. Firstly, Other Opening-up Policies: represented by the dummy variable of the Pilot Free Trade Zone ($FTZ_{ij}$). If the province i has become a pilot province of the Pilot Free Trade Zone in year j, the value is 1. In addition to the Belt and Road Initiative, the Pilot Free Trade Zone is also an important policy of China to promote opening-up, the dummy variable of the Pilot Free Trade Zone is added to test the heterogeneous impact of other opening-up policies on residents’ health level. Secondly, Economic Scale: represented by the natural logarithm of the GDP of each province (LnGDP). Thirdly, Income Level: represented by the natural logarithm of the average wages of on-the-job workers in urban units in each province (Lnincome). Fourthly, Education Level: represented by the proportion of college education level or above among employed persons in each province (Education). Fifthly, Urbanization Level: represented by the ratio of the urban residents’ population to the total residents’ population in each province (City). Sixthly, Unemployment Level: represented by the registered urban unemployment rate in each province (Unemployment). Seventhly, Medical Resources: the natural logarithm of the number of health personnel (Employees working in hospitals, primary medical and health institutions, professional public health institutions and other medical and health institutions, including health technicians, rural doctors and health workers, other technical personnel, administrative personnel and laborers) in each province (Lnpeople) and the number of beds in medical and health institutions per thousand people (Bed) in each province are used to represent the availability of medical human resources and medical bed resources. Eighthly, Population Aging Level: represented by the dependency ratio of the elderly population in each province (Old) 

### 2.4. Data Sources

The data used in this article is the provincial panel data of China from 2009 to 2020 (excluding Tibet, Hong Kong, Macau and Taiwan due to missing data). The relevant raw data are mainly from the *China Statistical Yearbook*, *China Environmental Statistical Yearbook* and *China Health Statistical Yearbook*. The descriptive statistical analysis of the main variables is shown in Table 2.

## 3. Results

### 3.1. Regional Inequality of Residents’ Health Level in China

With regional economic development and the continuous improvement of the public medical and health system, the health level of Chinese residents has been steadily improved. The national average Health Index of residents has increased from 0.609 in 2009 to 0.827 in 2020, with the residents’ health level increasing by 35.86%.

**Figure 1:**
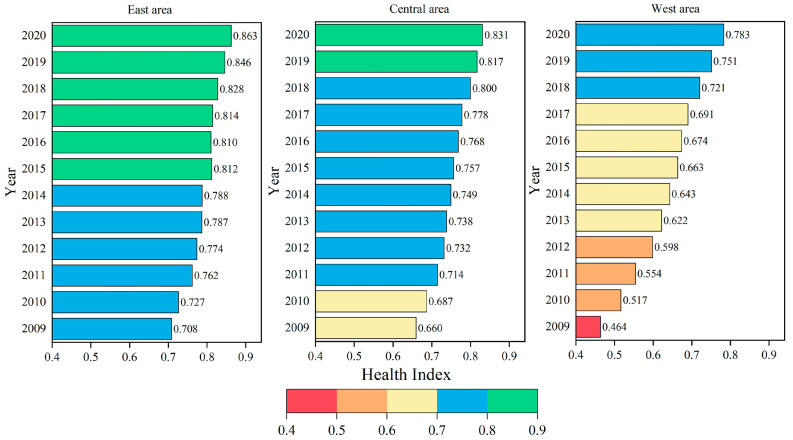
Change trends of regional Health Index in China from 2009 to 2020.

The regional changes of residents’ health level are further analyzed. Figure 1 shows the change trend of the Health Index in three major areas of East, Central and West areas in China from 2009 to 2020. Firstly, the East area enjoys a developed economy, a high degree of opening-up level and relatively complete medical and health infrastructure construction, its residents’ health level has been at a high level, but the growth rate has been relatively slow in recent years. Its Health Index has climbed from 0.708 in 2009 to 0.863 in 2020, with the residents’ health level only increasing by 21.82%. Secondly, the residents’ health level of the Central area has steadily improved. From 2009 to 2020, its Health Index increased from 0.660 to 0.831, with the residents’ health level increasing by 26.00%. Thirdly, it is worth noting that with the in-depth implementation of the Western Development Strategy and the Belt and Road Initiative in recent years, the West area has carried out in-depth exchanges and cooperation in the field of medical technology and health in China and abroad. Therefore, its residents’ health level has been greatly improved. From 2009 to 2020, the Health Index in the West area has surged from 0.464 to 0.783, with the residents’ health level increasing by 68.87%.

The difference in regional economic scale and the endowment of medical and health resources has caused the gap in residents’ health level between East and Central and West areas, while this gap is gradually narrowing. Specifically, in 2009, the Health Index of residents in the East area was 0.708, which was 0.049 and 0.245 higher than that in the Central and West areas, respectively. It can be found that the gap in health level of residents between the East and West areas was particularly obvious. While by 2020, the East area was only 0.032 and 0.080 ahead of the Central and West areas in terms of Health Index of residents, respectively.

**Figure 2:**
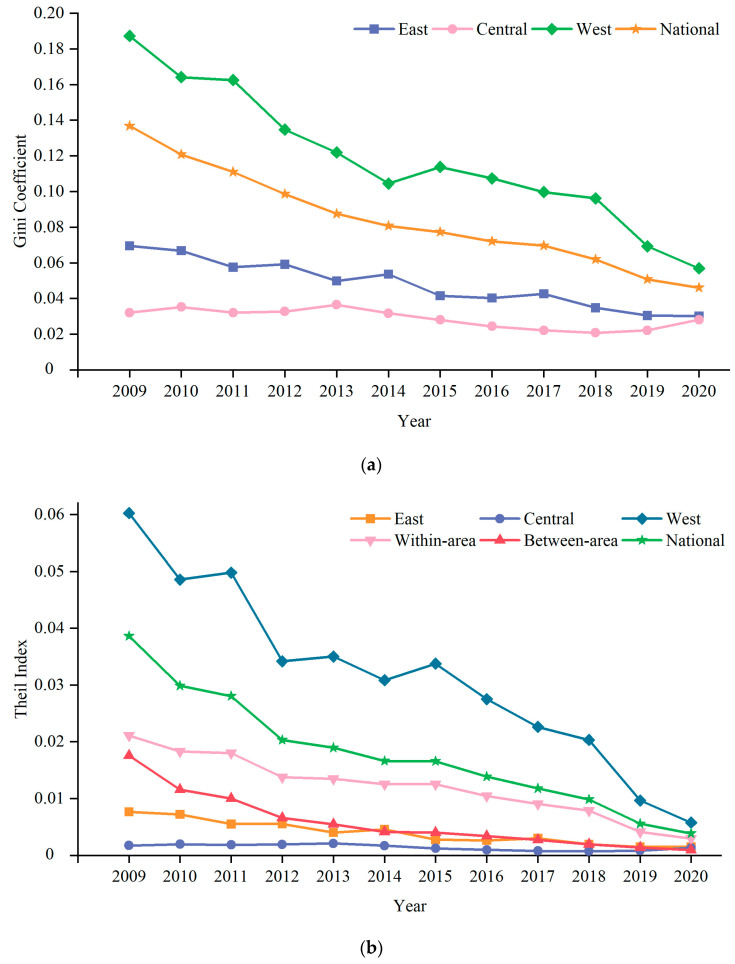
Regional inequalities in the health level of residents in China from 2009 to 2020 ((**a**): Gini Coefficient; (**b**): Theil Index).

The changing trends of regional health level of residents are further analyzed by the Gini Coefficient and Theil Index (Figure 2). It can be found that from 2009 to 2020, the inequality of the health level of Chinese residents among provinces changed greatly, with an overall trend of narrowing. The Gini Coefficient dropped from 0.1368 to 0.0460, with a decrease of 66.35%, and the Theil Index declined from 0.0386 to 0.0039, with a decrease of 89.98%. The Theil Index within three major areas proves that the overall inequalities in the health level of residents in China were mainly caused by inequalities within three major areas, with an average contribution rate of 71.09%. In general, the variation trends of the inequality in the health level of residents within three major areas and between three major areas were consistent with the overall variation trend, and all of them were steadily decreasing. It is worth noting that there were great differences in the variation of health level of the residents within three major areas. The inequalities in the residents’ health level in the East and Central areas remained relatively stable, while the West area witnessed a rapid narrowing trend. The Gini Coefficient of the West area decreased by 69.6% from 0.1873 to 0.0569, and the Theil Index of the West area slumped by 90.44% from 0.0602 to 0.0058. This indicated that the narrowing of the inequalities in the health level of residents in China was mainly attributed to the decline of the inequality in the provinces of the West area.

### 3.2. Spatial Distribution of Residents’ Health Level in China

**Figure 3:**
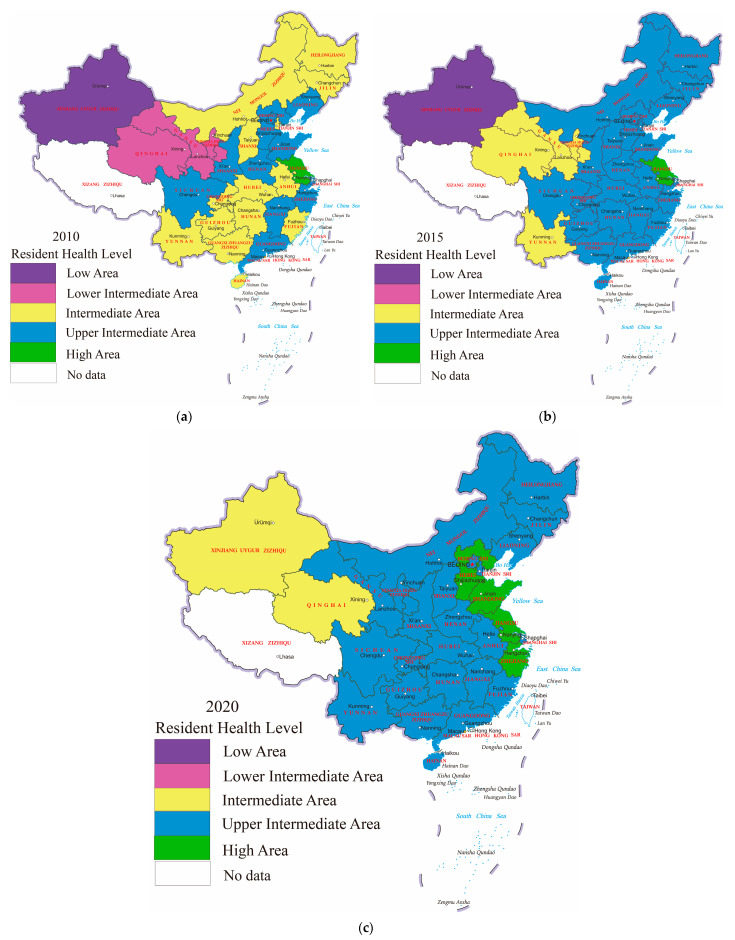
Spatial distribution patterns of residents’ health level in China ((**a**): 2010; (**b**): 2015; (**c**): 2020).

In order to further analyze the spatial distribution pattern of residents’ health level in China, based on the value of the Health Index of residents, the health level of residents is divided into five tiers. Specifically, Low Area (0.1–0.3), Lower Intermediate Area (0.3–0.5), Intermediate Area (0.5–0.7), Upper Intermediate Area (0.7–0.9) and High Area (>0.9). Figure 3 plots the spatial distribution characteristics and variation trend of residents’ health level in 2010, 2015 and 2020. It can be found that the spatial pattern of the health level of residents in China has changed greatly from 2010 to 2020. Firstly, from 2010 to 2015, 53.3% of the provinces transferred to higher health tiers and 46.7% of the provinces remained at original health tiers. To be specific, three provinces (Qinghai, Gansu and Ningxia) transferred from the Lower Intermediate Area to the Intermediate Area, and 13 provinces (Heilongjiang, Jilin, Inner Mongolia, Tianjin, Shanxi, Anhui, Hubei, Hunan, Chongqing, Guizhou, Guangxi, Fujian and Hainan) transferred from the Intermediate Area to the Upper Intermediate Area. One province (Xinjiang) remained at the Low Area, one province (Yunnan) remained at the Upper Intermediate Area, 11 provinces (Liaoning, Hebei, Beijing, Henan, Shandong, Shaanxi, Sichuan, Jiangxi, Zhejiang, Shanghai and Guangdong) remained at the Upper Intermediate Area and one province (Jiangsu) remained at the High Area. It can be found that the health level of residents in East and Central areas had been significantly improved from 2010 to 2015, which was closely related to the increase of medical and health technology and the rapid improvement of environmental quality in provinces of East and Central areas. The health level of residents in the West area had been improved to some extent, but its gap with the East and Central areas was still obvious. Especially in Xinjiang, the Health Index of residents only climbed from 0.204 in 2010 to 0.239 in 2015, and the health level of residents only increased by 17.03%. It was closely related to the fact that Xinjiang is an inland province, whose degree of opening-up was low, and there were few opportunities for medical and health technology exchanges and cooperation. 

Secondly, from 2015 to 2020, 23.3% of the provinces transferred to higher health tiers and 76.7% of the provinces remained at original health tiers. To be specific, one province (Xinjiang) achieved leapfrog development from a Low Area to an Intermediate Area, three provinces (Gansu, Ningxia and Yunnan) transferred from the Intermediate Area to the Upper Intermediate Area, three provinces (Hebei, Zhejiang and Shandong) transferred from the Upper Intermediate Area to the High Area. One province (Qinghai) remained at the Intermediate Area, one province (Jiangsu) remained at the High Area, and the remaining 21 provinces were all maintained at the Upper Intermediate Area. It can be concluded that the health level of residents in East and Central area had remained basically unchanged, while the health level of residents in provinces of West areas had been greatly improved. Particularly, the Health Index of residents in Xinjiang surged from 0.239 in 2015 to 0.649 in 2020, and the health level of residents improved by as much as 171.88%. It was closely related to the deepening of the Belt and Road Initiative in recent years, which has continuously promoted the improvement of the degree of opening-up and the active exchanges and cooperation in the field of medical and health care in the West area.

In general, the health level of residents in China witnessed a stable improve. From 2010 to 2015, the health level of residents in East and Central provinces improved significantly and then basically stabilized at the Upper Intermediate Area. From 2015 to 2020, the health level of residents in West provinces increased significantly and there is still great potential for the improvement of health level of residents in West areas. In addition, it can be found that the gap in the health level of residents between three major areas is reducing. The inequality within the East and Central provinces is no longer obvious, while the difference within the West provinces is still significant. Therefore, the reduction in the inequality in the health level of residents within the West provinces should be emphasized to further reduce the overall gaps in the health level of residents in China.

### 3.3. Influencing Factors of Residents’ Health Level in China

#### 3.3.1. Benchmark Regression

Table 3 shows the benchmark regression results of the impact of the opening-up policy on the health level of regional residents. In the benchmark regression, the coefficients of Treat×post are 0.041 and 0.042, respectively, which are both significant at the 1% level. The regression $adj.\;R^{2}$ are 93.6% and 93.8%, respectively, which proves the high explanation of the model in relation to the predicted data. It can be concluded that the Belt and Road Initiative has significantly improved the residents’ health level in the provinces along the route, which is closely related to the fact that the Belt and Road Initiative adheres to the development concept of the “Healthy Silk Road” and actively promotes exchanges and cooperations with countries and regions along the route in the field of medical and health care. Therefore, provinces along the route can take advantage to develop the medical and health industry and improve the level of medical technology. However, in terms of the Pilot Free Trade Zone, the coefficient of FTZ is 0.020, which is significant at the 1% level. Therefore, it has had an inhibitory effect on the health level of residents. It cannot be ignored that the provinces of the Pilot Free Trade Zone are mostly located in the developed East area; thus, the potential for the increase in the residents’ health level is small. However, in addition to the objective reasons, the main reasons for the Pilot Free Trade Zone to inhibit the regional health level of residents are the neglect of the development of the medical and health advantage industries in the zone and the failure to make full use of the dividends of opening-up to further enhance the soft environment of regional development.

The influence of the control variables on residents’ health level is further considered. Firstly, the coefficient of LnGDP is significantly negative, which is mainly because the growth of economic scale may cause regional environmental pollution and, at the same time, bring difficulties to disease prevention and inhibit the development and progress of the medical and health industry and technology, thereby reducing the health level of residents. It also proves that the simple growth of economic scale cannot promote the improvement of residents’ health to a certain extent, and efforts should be made to promote the high-quality development of the regional economy, instead of harming the regional environment and residents’ health in exchange for rapid economic development [23]. Secondly, the coefficient of Lnincome is significantly positive, which is mainly because the improvement of income level can promote residents to increase their investment in personal health, so as to improve the health level of residents [24]. In addition, it can be found that compared with the growth of regional economic scale, the increase in residents’ income level is a more effective way to improve the health level of residents. Thirdly, the coefficient of Education is significantly positive, which is mainly since the improvement of education level can promote residents to form a correct life and behavior style, enhance awareness of personal health and disease prevention, so as to improve their health level [25]. Fourthly, the coefficient of City is significantly positive, which is mainly because the city has a more complete medical and health system, and residents have more access to advanced and convenient medical facilities and services. Therefore, the improvement of the urbanization level can promote the improvement of regional residents’ health [25]. Fifthly, the coefficient of Unemployment is significantly negative, which is mainly because unemployment will cause the loss of income source, and it is easy to produce negative psychological emotions and develop bad living habits, thereby reducing the health level of residents [26]. Sixthly, the coefficient of Lnpeople is significantly positive, while the coefficient of Bed is significantly negative. This is mainly because the increase in the number of health personnel can improve the service capacity of medical institutions and thus, improve the health level of residents. However, the increase in the number of medical beds may have the problem of low utilization of medical beds, which cannot play a positive role in improving the health level of residents [27]. Seventhly, the coefficient of Old is significantly negative, which is mainly since the aging will cause the decline of the immunity of body, and coupled with the fact that older adults often have more chronic diseases; thus, the risk of infectious diseases is significantly increased [28].

#### 3.3.2. Parallel Trend Test

**Figure 4:**
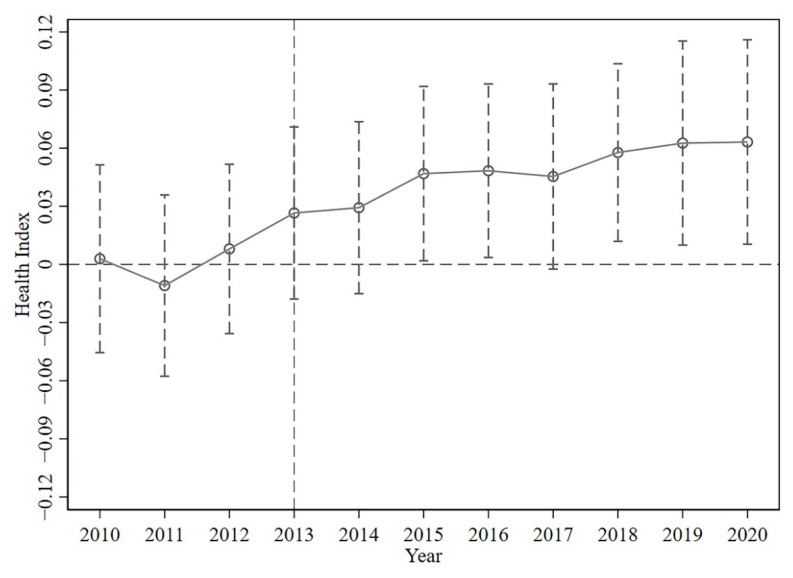
Parallel trend test.

The premise of using the Difference-in-Difference model is to satisfy the parallel trend hypothesis. The result of the benchmark regression has proven that the Belt and Road Initiative has significantly increased the residents’ health level in the provinces along the route. To further confirm the robustness of the benchmark regression result, it is necessary to verify whether there is a significant difference in the residents’ health level among provinces which are along the route of the Belt and Road Initiative and provinces which are not along the route. If there is no significant difference in the Health Index between the treatment group and the control group before the put forward of the Belt and Road Initiative, the parallel trend assumption is satisfied. In this section, the treatment time dummy variables in Equation (6) are replaced by the dummy variables of the sample years from 2009 to 2020 and the regression is performed with 2009 as the benchmark period. It can be found from Figure 4 that before the put forward of the Belt and Road Initiative, there was no significant difference in the Health Index between the treatment group and the control group. Therefore, the parallel trend hypothesis is satisfied, confirming the reliability of the result obtained in the benchmark regression.

The dynamic policy effects of the Belt and Road Initiative are further analyzed. It can be found that after the put forward of the Belt and Road Initiative, although the Health Index of the provinces along the route has increased obviously, it was not significant at the 5% level. Only since 2015, the policy effect has passed the 5% significance level test. On the one hand, in March 2015, China officially released the *Vision and Actions on Jointly Building Silk Road Economic Belt and 21st-Century Maritime Silk Road*, which marks the full launch of the Belt and Road Initiative. In addition, in 2015, the National Health and Family Planning Commission of China issued the *Three-year Implementation Plan on Promoting Health Exchanges and Cooperation along the Belt and Road (2015–2017)*, officially launching the construction of the “Healthy Silk Road” and promoting exchanges and cooperation with countries and regions along the route in the field of health care. Therefore, since 2015, the health effects of the Belt and Road Initiative have begun to manifest. On the other hand, compared with the growth of regional economic scale and the improvement of regional environmental quality, the increase in residents’ health is a long process. Although China has actively promoted exchanges and cooperation in the field of medical and health care with countries and regions along the route since the Belt and Road Initiative was proposed in 2013, the promotion effect of the Belt and Road Initiative cannot be immediate. Therefore, in the first few years of the Belt and Road Initiative, it failed to give full play to the promotion of the health level of residents in the provinces along the route, but with the passage of time, its health effect has gradually appeared. In addition, considering the influence of some unforeseen problems like the COVID on opening-up and residents’ health, it can be found that in 2020, when it was the first year of the pandemic, the Belt and Road Initiative still had obvious effects on the improvement of residents’ health.

#### 3.3.3. Placebo Test

**Figure 5:**
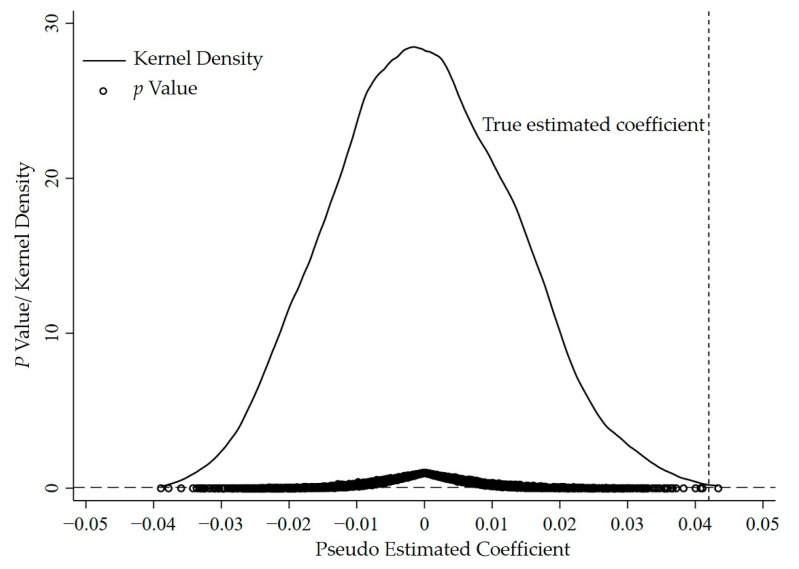
Placebo test.

When using the Difference-in-Difference model, some unobservable but time-varying provincial characteristics may have an impact on the result of the benchmark regression. Each province does have different characteristics, but it is not practical to add all control variables that might affect the result of the benchmark regression. Therefore, the placebo test is used to avoid the interference of the benchmark regression result due to unobserved or omitted variables [29]. Specifically, the research sample includes 30 provinces in China from 2009 to 2020, of which 17 provinces are along the route of the Belt and Road Initiative. Firstly, 17 provinces are randomly selected from the sample of 30 provinces as the pseudo treatment group. Then, one year is randomly selected from 2009 to 2020 as the pseudo treatment time for each pseudo treatment province. Finally, 3000 times of the pseudo regressions are performed on this basis. Figure 5 plots the regression results of the placebo test. It can be found that the simulation-estimated coefficients are concentrated around 0 and approximately obey the normal distribution, and most of the simulated estimated coefficients are not significant at the 5% level. In addition, it can be found that the coefficient of the core explanatory variable in the benchmark regression is significantly different from the pseudo estimated coefficients of the treatment group and the treatment time randomly selected. Therefore, it can be concluded that the benchmark regression model has passed the placebo test and the interference of the benchmark estimation result by unobservable variables or omitted variables can be excluded.

#### 3.3.4. Mechanism Analysis

The previous results have shown that the Belt and Road Initiative has significantly promoted the improvement of the health level of residents in the provinces along the route. In this section, its theoretical mechanisms are further analyzed.

Firstly, the Belt and Road Initiative improves the health of residents in the provinces along the route by increasing the degree of regional opening-up. The Belt and Road Initiative is an important measure for China to promote opening-up, which is an important platform for provinces along the route in China to carry out international medical technology cooperation and joint training of medical talents [30]. The domestic medical, health and elderly care markets are still immature in China. On the one hand, the improvement of the degree of opening-up will enable excellent foreign medical and elderly care companies to enter. On the other hand, it can strengthen exchanges and cooperation with other countries and regions in the field of medical and health care. Therefore, more advanced medical and health concepts and technologies can be provided, the supply of high-quality health services can be increased and the residents’ health levels can be increased [31].

Secondly, the Belt and Road Initiative improves the health of residents in provinces along the route by improving the regional environmental quality. Environmental quality is an important factor affecting the health level of residents [32,33]. It has been found that there was a significant negative correlation between air pollution level and residents’ health level [34]. It has been confirmed that there is a significant inverse relationship between clean energy use and maternal mortality [8]. The Belt and Road Initiative attaches importance to environmental protection and green development, and is committed to building a “Green Silk Road” and improving the environmental quality of provinces along the route [35,36]. Therefore, the Belt and Road Initiative can increase the residents’ health levels by improving environmental quality and creating a healthy living environment of provinces along the route.

Based on the above analysis, the moderating effect model is used to test the mechanism of the Belt and Road Initiative to improve the health level of residents in the provinces along the route. Regarding the effect of the increase in the degree of opening-up, the natural logarithm of the actual use of foreign direct investment in each province (LnFDI) is selected as the moderating variable to measure the degree of opening-up of each province. The specific model is shown as follows.

(7) $$Health_{ij}=\beta_{0}+\beta_{1}Treat_{i}\times post_{j}\times LnFDI_{ij}+\gamma X_{ij}+\alpha_{i}+\delta j+\varepsilon_{ij}$$

Regarding the effect of the improvement of the regional environmental quality, the entropy method is utilized to calculate the Environment Quality Index (Environment) to measure the regional environmental quality. Firstly, based on industrial solid waste generation, sulfur dioxide emissions and total wastewater emissions, the data is processed by reverse standardization. Then, the entropy method is used to determine the weight of each indicator. Finally, the comprehensive score is obtained by linear weighting. Therefore, the Environment Quality Index in each province is obtained to represent the regional environmental quality level. The specific model is shown as follows.

(8) $$Health_{ij}=\beta_{0}+\beta_{1}Treat_{i}\times post_{j}\times Environment_{ij}+\gamma X_{ij}+\alpha_{i}+\delta_{j}+\varepsilon_{ij}$$

Table 4 reports the regression results of the mechanism test. Column (1) is the regression result of the effect of the increase in the degree of opening-up, the regression coefficient of is 0.002, which passes the significance level test of 1%. Column (2) is the regression result of the effect of the improvement of the regional environmental quality, the regression coefficient of is 0.054, which also passes the significance level test of 1%. The above results indicate that the policy effect of the Belt and Road Initiative on the residents’ health levels is moderated by the degree of opening-up and the regional environmental quality. The higher the degree of the opening-up and the better the regional environmental quality, the greater the promotion effect of the Belt and Road Initiative on the improvement of the health level of residents in the provinces along the route.

## 4. Discussion

Through the analysis, the regional health inequalities and the impacts of opening-up policies and other factors on residents’ health have been explored. However, there are still some limitations existing in the following aspects. 

Firstly, in order to divide the overall differences in residents’ health in China into differences within three major areas and differences between three major areas, the Theil Index is used. However, as a back-up to the main findings of the inequalities of residents’ health, the Theil Index is a lack of rigor, to some extent. Since it is a typically measuring indicator for economic inequality. In a future study, more measurement methods like kernel density and variable coefficient could be utilized.

Secondly, due to the limitation of the time spans of data, the influence of some unforeseen problems like the COVID-19 pandemic on opening-up and residents’ health cannot be analyzed precisely. Therefore, this interesting and important topic will be further studied in the future.

Thirdly, from a global perspective, the development of opening-up can have several negative impacts, including on health. This is not common in China but might occur. Therefore, the reasons for the different impacts of opening-up on residents’ health in China and other countries are supposed to be analyzed in a future study.

In addition, focusing on increasing residents’ health levels and reducing regional health inequalities, suggestions from the following three aspects are provided.

Firstly, the role of the Belt and Road Initiative as a platform for exchanges and cooperation with countries and regions along the route in the field of medical and health care should be fully utilized. The connection with countries and regions along the route in terms of medical talents and technology, traditional Chinese medicine exchange and promotion and major public health emergencies monitoring and prevention should be improved. The development of health advantage industries such as health service trade and health medical tourism should be supported, the participation of the society and enterprises in terms of the cooperation in the field of medical and health care under the Belt and Road Initiative is supposed to be encouraged, and an all-round, multi-level, wide-ranging medical and health cooperation network under the topic of “Healthy China 2030” should be constructed.

Secondly, the release of the development dividends of the Pilot Free Trade Zone in the field of medicine and health reform needs to be accelerated, the optimization of health-related approval items in the zone is supposed to be promoted, and the development models and institutional mechanisms should be innovated. It is necessary to further attract foreign high-quality medical and health enterprises to settle in the Pilot Free Trade Zone, so as to build medical and health industries into advantageous industries and promote the high-quality development of the health industry in the zone.

Thirdly, in response to the inequalities of residents’ health levels in different regions, a path for the equitable development of residents’ health should be constructed. The dividends of opening-up should be further utilized, the development between urban and rural areas and among regions is supposed to be coordinated, and the integration of environmental facilities and medical security systems between urban and rural areas and among regions should be promoted. In addition, the government is supposed to introduce preferential policies for medical security for people with high disease incidence, thereby promoting the effective implementation of the “Healthy China 2030” strategy and leading the global environmental health governance. 

## 5. Conclusions

Based on provincial panel data of China from 2009 to 2020, the Gini Coefficient and the Theil Index are used to analyze the regional inequalities in residents’ health levels in China. On this basis, the Difference-in-Difference model is constructed to study the impact of China’s opening-up policies and other factors on residents’ health levels. The main conclusions of the research are provided as follows.

Firstly, from 2009 to 2020, the health level of Chinese residents has steadily improved, especially in the West area. The variation trends of the inequality in the health level of residents within three major areas and between three major areas were consistent with the overall variation trend, and all of them were steadily decreasing. The overall inequalities in the health level of residents in China were mainly caused by inequalities within three major areas, with an average contribution rate of 71.09%, and the narrowing of the inequalities in the health level of residents in China was mainly attributed to the decline of the inequality in the provinces of the West area.

Secondly, from the perspective of the impact of the opening-up policies in China on the residents’ health level, the Belt and Road Initiative has significantly improved the residents’ health level in the provinces along the route, and this promotion effect has only been apparent since 2015. However, in terms of the Pilot Free Trade Zone, which is another important opening-up policy in China, has had an inhibitory effect on the health level of residents. It cannot be ignored that the provinces of the Pilot Free Trade Zone are mostly located in the developed East area; thus, the potential for the increase in the residents’ health levels is small. However, in addition to the objective reasons, the main reasons for the Pilot Free Trade Zone to inhibit the regional health level of residents are the neglect of the development of the medical and health advantage industries in the zone, and the failure to make full use of the dividends of opening-up to further enhance the soft environment of regional development.

Thirdly, comprehensively considering other factors influencing the health level of residents in China, the increase in residents’ income level, education level, urbanization level and the number of health personnel can improve the health level of residents. On the contrary, the growth of the economic scale, the increase in the number of medical beds, the unemployment rate and the aging of the population have a significant inhibitory effect on the health level of residents.

Fourthly, the mechanism analysis shows that the Belt and Road Initiative improves the health of residents in provinces along the route by increasing the degree of opening-up and improving the regional environmental quality.

## Figures and Tables

**Table 1:** Evaluation index system of residents’ health level.

First-Level Indicator	Second-Level Indicator	Indicator Direction	Indicator Weight
Residents’ Health Level	Child (in Perinatal) Mortality Rate	Negative	0.25
	Maternal Mortality Rate	Negative	0.29
	Infectious Diseases Morbidity Rate	Negative	0.28
	Gynecological Diseases Detection Rate	Negative	0.18

**Table 2:** Descriptive statistical analysis of main variables.

Variable	Observations	Mean	Std. Dev	Min	Max
Health	360	0.729	0.138	0.187	0.953
FTZ	360	0.194	0.396	0	1
LnGDP	360	9.725	0.891	6.986	11.615
Lnincome	360	10.945	0.398	10.114	12.128
Education	360	9.124	0.908	7.034	12.701
City	360	57.550	12.935	29.890	93.768
Unemployment	360	3.322	0.647	1.210	4.610
Lnpeople	360	12.538	0.734	10.447	13.843
Bed	360	5.029	1.243	2.510	7.950
Old	360	14.166	3.579	7.440	25.480

**Table 3:** Benchmark regression result. Note: Column (1) is the regression result only considering the opening-up policy of the Belt and Road Initiative and column (2) is the regression result further considering other opening-up policies, namely Pilot Free Trade Zone. *, ** and *** denote statistical significance at the 10%, 5% and 1% level, respectively. Numbers in brackets are robust standard errors.

Variable	(1)	(2)
	Health	Health
Treat×post	0.041 ***	0.042 ***
	(0.009)	(0.009)
FTZ		−0.020 ***
		(0.007)
LnGDP	−0.080 ***	−0.070 ***
	(0.023)	(0.023)
Lnincome	0.126 **	0.115 *
	(0.063)	(0.062)
Education	0.027 *	0.026 *
	(0.014)	(0.014)
City	0.008 ***	0.008 ***
	(0.002)	(0.002)
Unemployment	−0.026 ***	−0.024 ***
	(0.009)	(0.008)
Lnpeople	0.115 *	0.111 *
	(0.067)	(0.067)
Bed	−0.016 **	−0.015 **
	(0.007)	(0.006)
Old	−0.004 *	−0.004 *
	(0.002)	(0.002)
_cons	−1.843 *	−1.736 *
	(0.982)	(0.973)
Provincial Fixed Effect	YES	YES
Year Fixed Effect	YES	YES
Observations	360	360
$adj.\;R^{2}$	0.936	0.938

**Table 4:** Mechanism test result. Note: Column (1) is the regression result of the effect of the increase in the degree of opening-up, Column (2) is the regression result of the effect of the improvement of the regional environmental quality. * and *** denote statistical significance at the 10% and 1% level, respectively. Numbers in brackets are robust standard errors.

Variable	(1)	(2)
	Health	Health
Treat×post×LnFDI	0.002 ***	
	(0.001)	
Treat×post×Environment		0.054 ***
		(0.011)
_cons	−1.830 *	−1.602
	(0.964)	(0.983)
Control Variables	YES	YES
Provincial Fixed Effect	YES	YES
Year Fixed Effect	YES	YES
Observations	360	360
$adj.\;R^{2}$	0.935	0.938

## Data Availability

Some or all data and models that support the findings of this study were available from the corresponding author upon reasonable request.
